# Supplementary material for: The Grb2 splice variant, Grb3-3, is a negative regulator of RAS activation
Source: Commun Biol. 2022 Sep 28;5:1029. doi: 10.1038/s42003-022-03985-7 (PMC9519967; doi:10.1038/s42003-022-03985-7)
Supplement: Supplementary file 2 — Supplementary Material [file 42003_2022_3985_MOESM2_ESM.pdf]

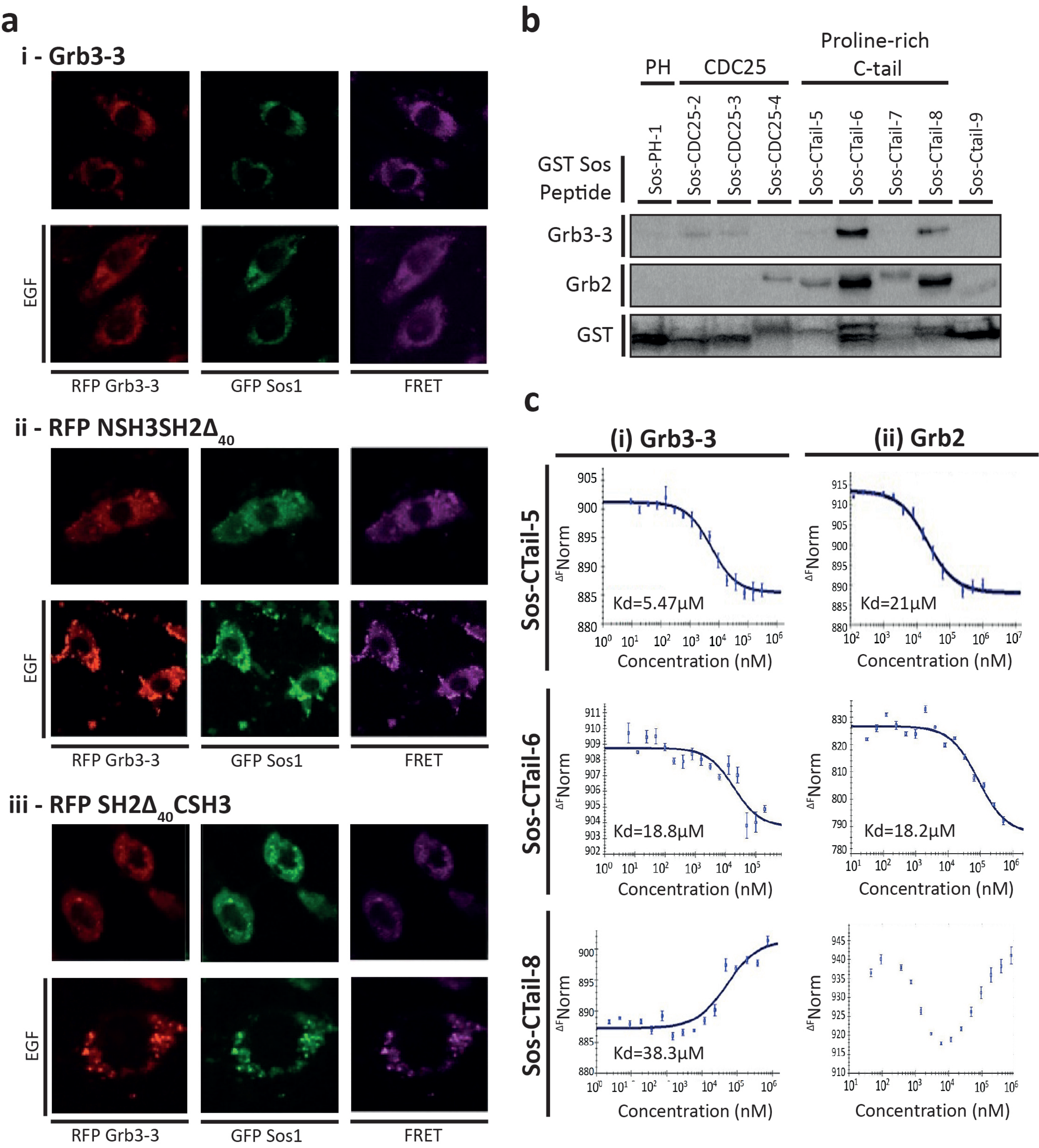

a
i - Grb3-3
EGF
RFP Grb3-3
GFP Sos1
FRET
ii - RFP NSH3SH2Δ40
EGF
RFP Grb3-3
GFP Sos1
FRET
iii - RFP SH2Δ40CSH3
EGF
RFP Grb3-3
GFP Sos1
FRET
b
PH
CDC25
Proline-rich C-tail
GST Sos Peptide
Sos-PH-1
Sos-CDC25-2
Sos-CDC25-3
Sos-CDC25-4
Sos-CTail-5
Sos-CTail-6
Sos-CTail-7
Sos-CTail-8
Sos-Ctail-9
Grb3-3
Grb2
GST
c
(i) Grb3-3
(ii) Grb2
Sos-CTail-5
Sos-CTail-6
Sos-CTail-8
ΔFNorm
Kd=5.47μM
Kd=21μM
Kd=18.8μM
Kd=18.2μM
Kd=38.3μM
Concentration (nM)

Supplementary Figure 1: Grb3-3 associates with the Sos C-terminal tail

(a) Fluorescence resonance energy transfer (FRET) images demonstrating the interaction between Grb3-3 and Sos in HEK293T cells stably transfected with fibroblast growth factor receptor 2 (FGFR2) under conditions of serum-starvation (top panels) and stimulation with epidermal growth factor (EGF). (i) FRET following co-transfection with RFP-tagged full-length Grb3-3 and GFP-tagged Sos. (ii) FRET following co-transfection with truncated RFP-tagged Grb3-3, comprising of its N-terminal SH3 and SH2 domains, (NSH3SH2Δ_{40}) and GFP-tagged Sos. (iii) FRET following co-transfection with truncated RFP-tagged Grb3-3, comprising of its C-terminal SH3 and SH2 domains (SH2Δ_{40}CSH3), and GFP-tagged Sos. Control cells transfected with RFP alone showed no FRET (not shown). Scale bar 10μm. **(b)** Western-blot demonstrating binding of purified His-tagged full-length Grb3-3 and full-length Grb2 to GST-tagged proline-rich peptides derived from the Sos C-terminal tail. The domains from which each Sos peptide sequence is derived are shown at the top of the chart (PH, pleckstrin homology). A full list of tail sequences can be found in **Supplementary Table 1.** Sos9 peptide (not included in **Supplementary Table 1**) corresponds to GST alone. Red arrows are used to highlight probable interactions. An interaction between Grb2 and Grb3-3 is seen with Sos-CTail peptides -5, -6 and -8. **(c)** MST measurement of the interaction between purified full-length His tagged (i) Grb3-3 (left column) and (ii) Grb2 (right column) with Sos-CTail proline-rich peptides -5, -6 and -8. Unlabelled peptides (10nM-1mM) were titrated into a fixed concentration (100nM) of labelled Grb2 or Grb3-3. These confirm binding of Grb2 and Grb3-3 to Sos-CTail peptides -5 and -6.

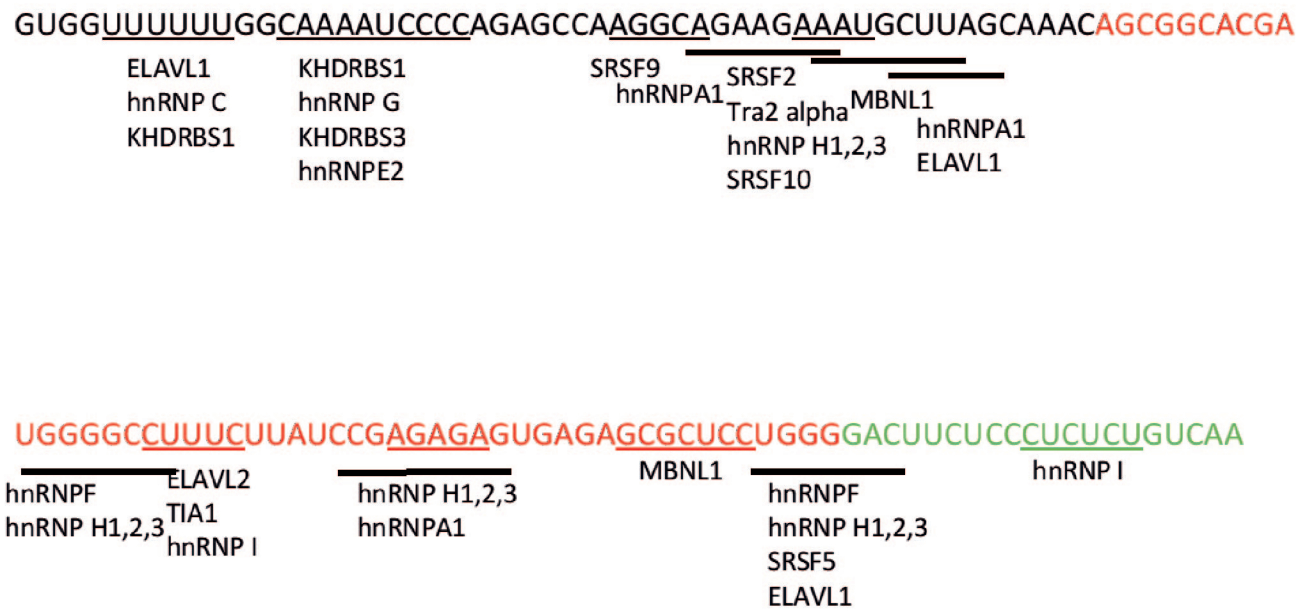
GUGGUUUUUUGGCAAAAUCCCCAGAGCCAAGGCAGAAGAAAUGCUUAGCAAACAGCGGCACGA
ELAVL1
hnRNP C
KHDRBS1
KHDRBS1
hnRNP G
KHDRBS3
hnRNPE2
SRSF9
hnRNPA1
SRSF2
Tra2 alpha
hnRNP H1,2,3
SRSF10
MBNL1
hnRNPA1
ELAVL1
UGGGGCCUUUCUUAUCCGAGAGAGUGAGAGCGCUCCUGGGGACUUCUCCCUCUCUGUCAA
hnRNPF
hnRNP H1,2,3
ELAVL2
TIA1
hnRNP I
hnRNP H1,2,3
hnRNPA1
MBNL1
hnRNPF
hnRNP H1,2,3
SRSF5
ELAVL1
hnRNP I

Supplementary Figure 2: Location of consensus sites for splicing factors predicted to bind to *GRB2* exon 4 by SpliceAid2

An *in silico* analysis using SpliceAid2 with the sequence for *GRB2* exon 4 as input revealed a number of potential transcription factors predicted to bind *GRB2* exon 4. A majority were predicted to bind early in the exon sequence. Putative transcription factor binders are aligned against the sequence to which they were predicted to bind.

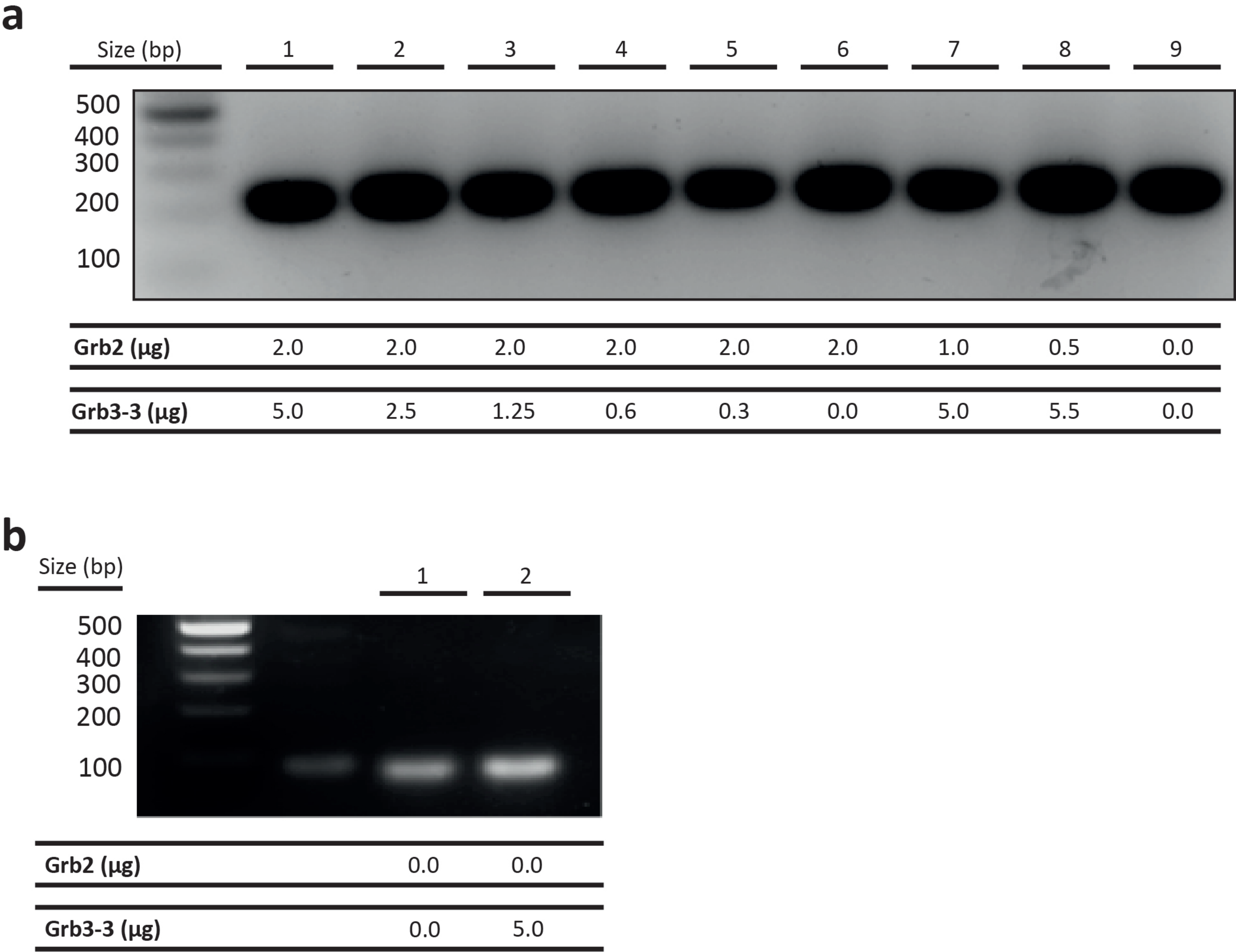
a
Size (bp)
1 2 3 4 5 6 7 8 9
500
400
300
200
100
Grb2 (µg) 2.0 2.0 2.0 2.0 2.0 2.0 1.0 0.5 0.0
Grb3-3 (µg) 5.0 2.5 1.25 0.6 0.3 0.0 5.0 5.5 0.0
b
Size (bp)
1 2
500
400
300
200
100
Grb2 (µg) 0.0 0.0
Grb3-3 (µg) 0.0 5.0

Supplementary Figure 3: Determining the specificity of isoform-specific sensitive primers for the splice-sensitive Grb2/Grb3-3 qRT-PCR assay.

Agarose gel electrophoresis was used to determine primer specificity for the splice-sensitive Grb2/Grb3-3 qRT-PCR assay, and to ensure there was no cross-reactivity between the two primer transcripts or mis-priming of non-specific genes. **(a)** To assess the specificity of the Grb3-3 primer pair, HeLa cells were transfected with 0-5μg exogenous Grb3-3 cDNA prior to RNA extraction. Although endogenous Grb2 expression is recognised in HeLa cells, co-transfection with 0-2.0μg Grb2 was undertaken prior to RNA extraction to ensure no cross-reactivity under conditions in which Grb2 concentration is high. Grb3-3 primers were used and the PCR product run via agarose gel electrophoresis. In all samples, a single band was produced between 200 and 300 base pairs (bp) in size, corresponding to the expected amplicon size of 223bp for the Grb3-3 primers. This band was seen in all samples, including those not transfected with exogenous Grb3-3 cDNA, indicating that the primers are sensitive enough to detect endogenous levels of the Grb3-3 transcript. The PCR fragment from sample 9, corresponding to endogenous Grb3-3, was gel extracted, cloned into a TA vector and sequenced confirming that the PCR product produced was Grb3-3. **(b)** To assess the specificity of Grb2-specific primers, the Grb2 primer pair was used to generate a PCR product from RNA extracted from HeLa cells. Given the known endogenous Grb2 expression in HeLa cells, no exogenous Grb2 cDNA was transfected prior to RNA extraction. In order to ensure there was no cross-reactivity with Grb3-3, one sample was transfected with 5μg of Grb3-3 cDNA. A single band was produced in both samples, indicating no cross reactivity. The band observed is less than 100 base pairs in size, corresponding to the expected amplicon size for the Grb2

primers of 76bp. Sequencing of the PCR product following gel extraction confirmed that the product was Grb2 mRNA.

Figure 1a

pERK

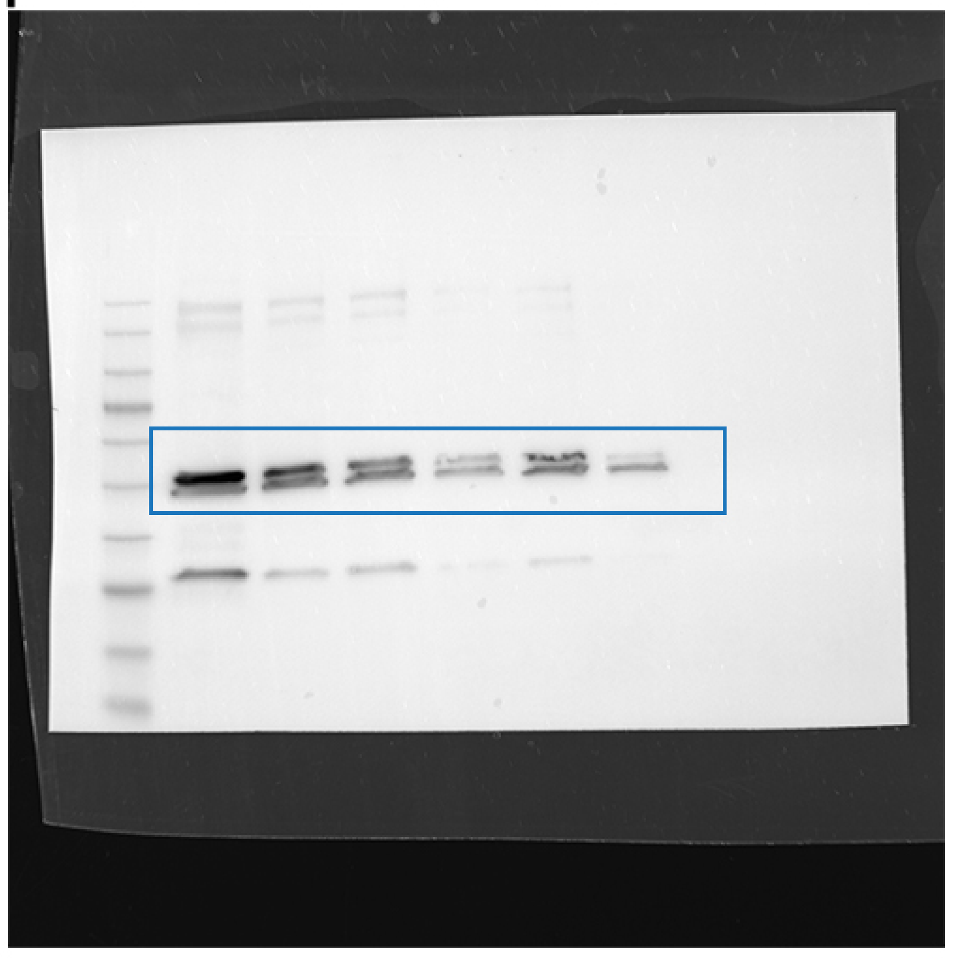

ERK

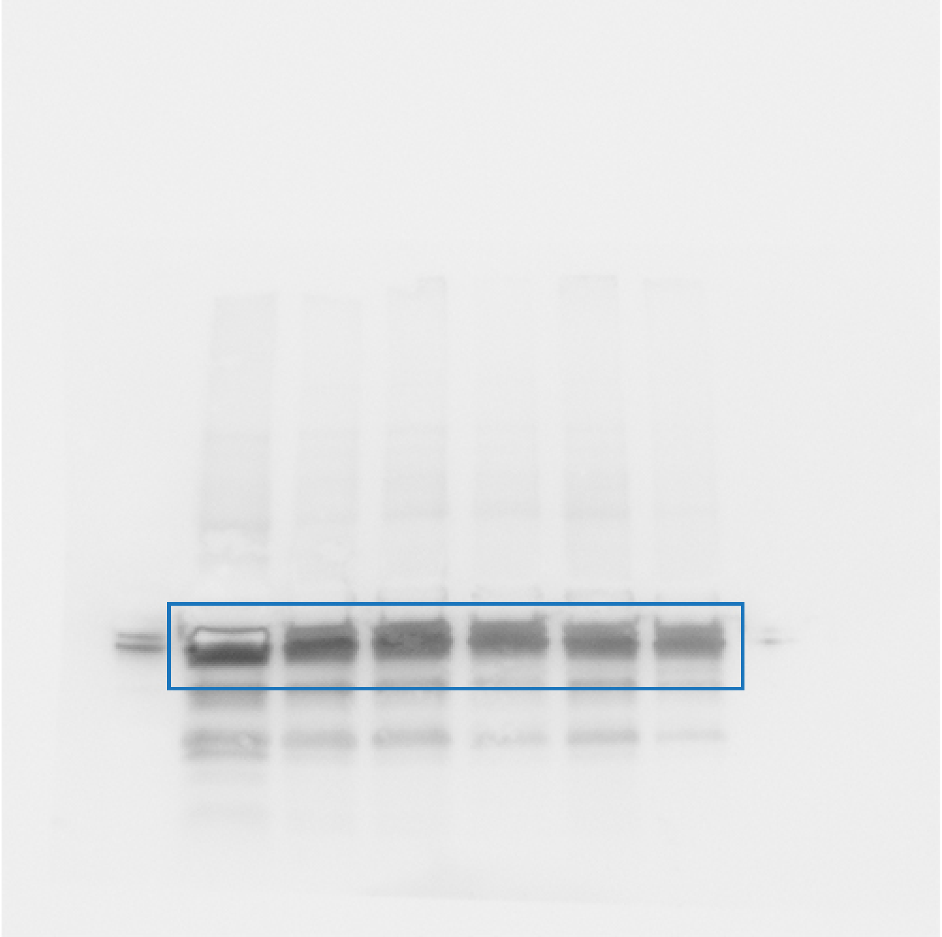

α-Tubulin

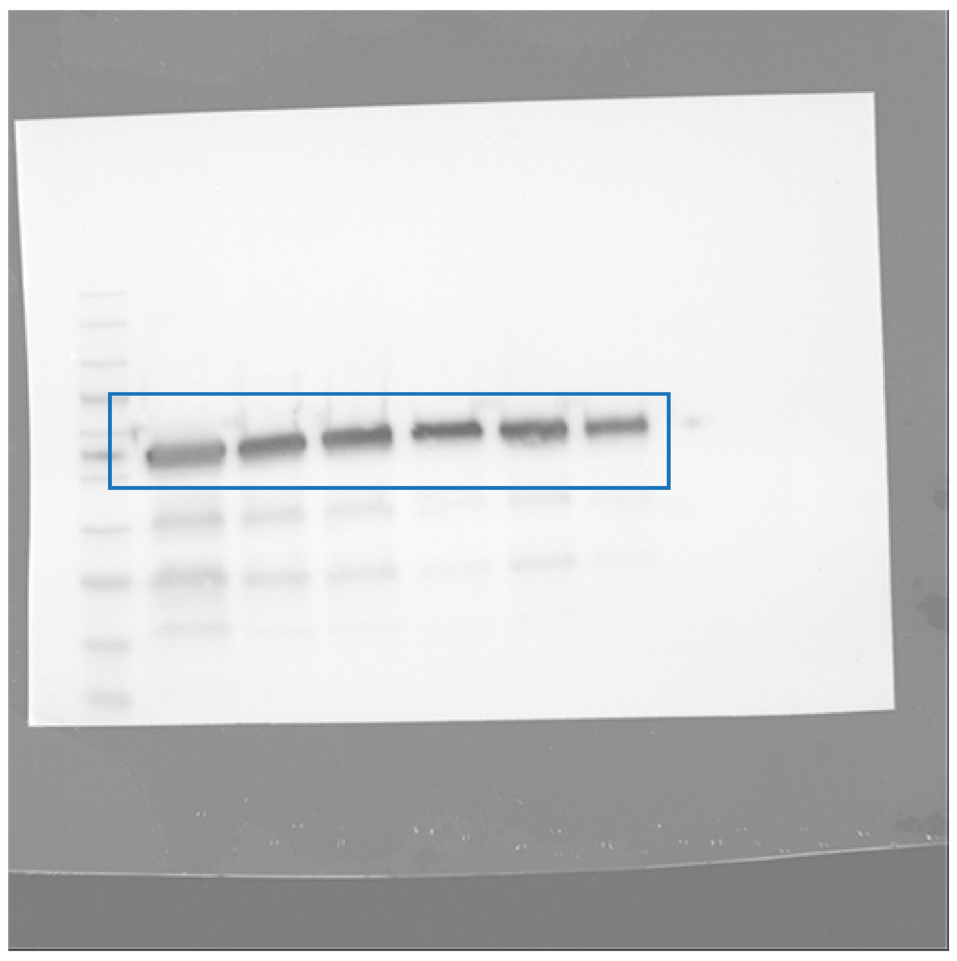

Figure 1c

pEGFR

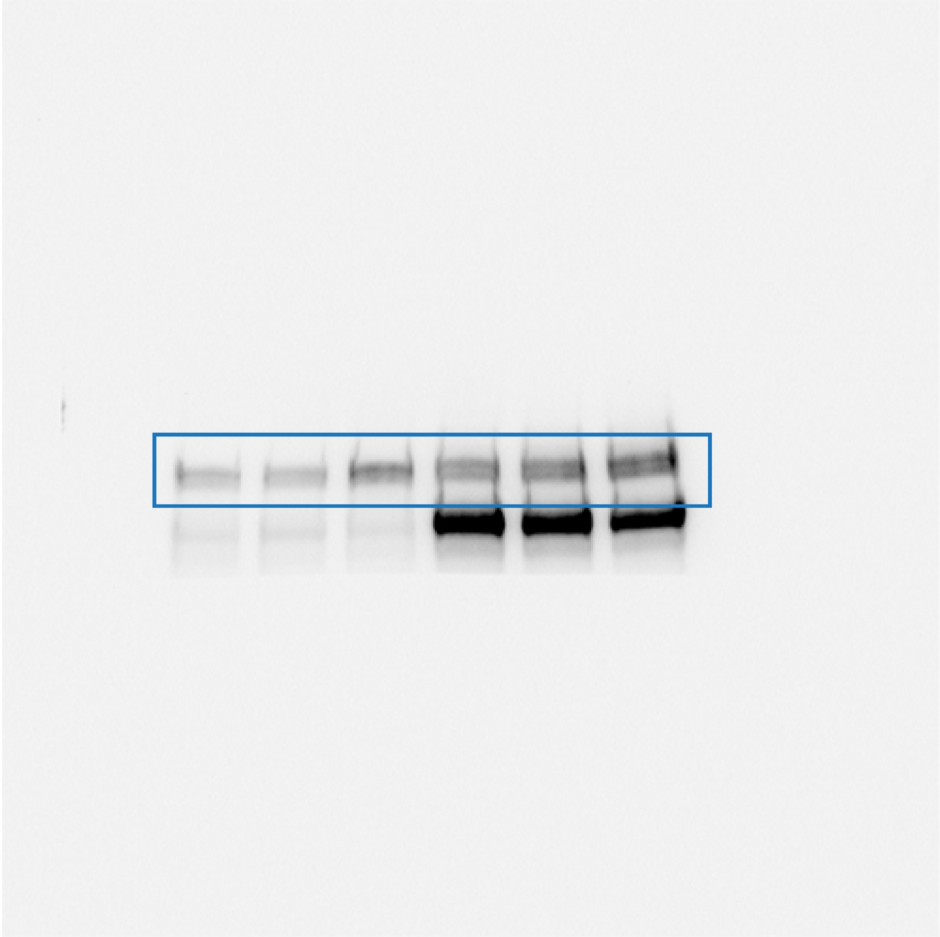

α-Tubulin

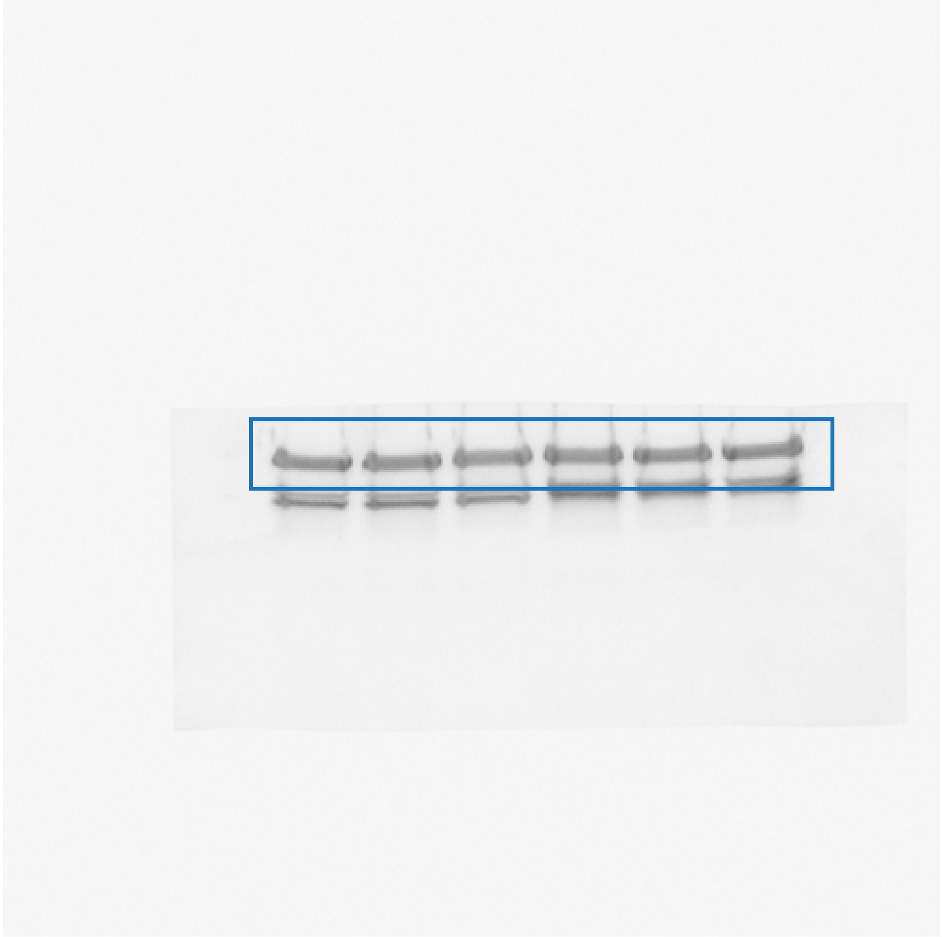

pERK

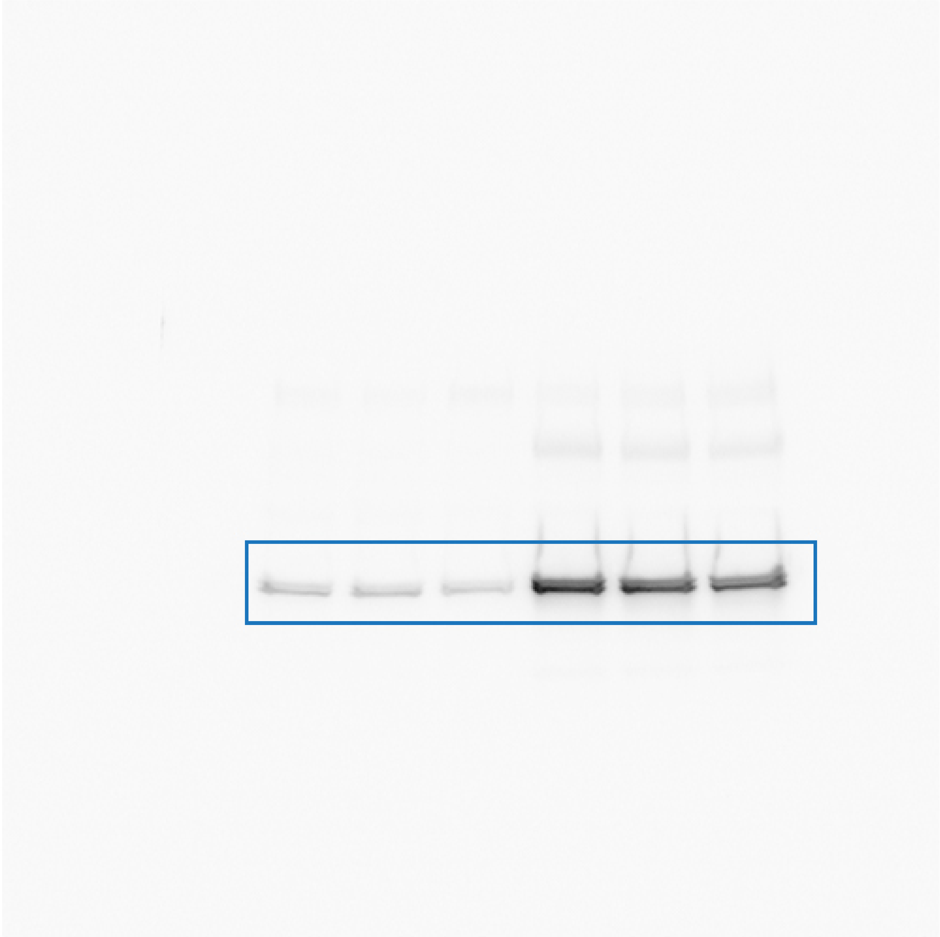

EGFR

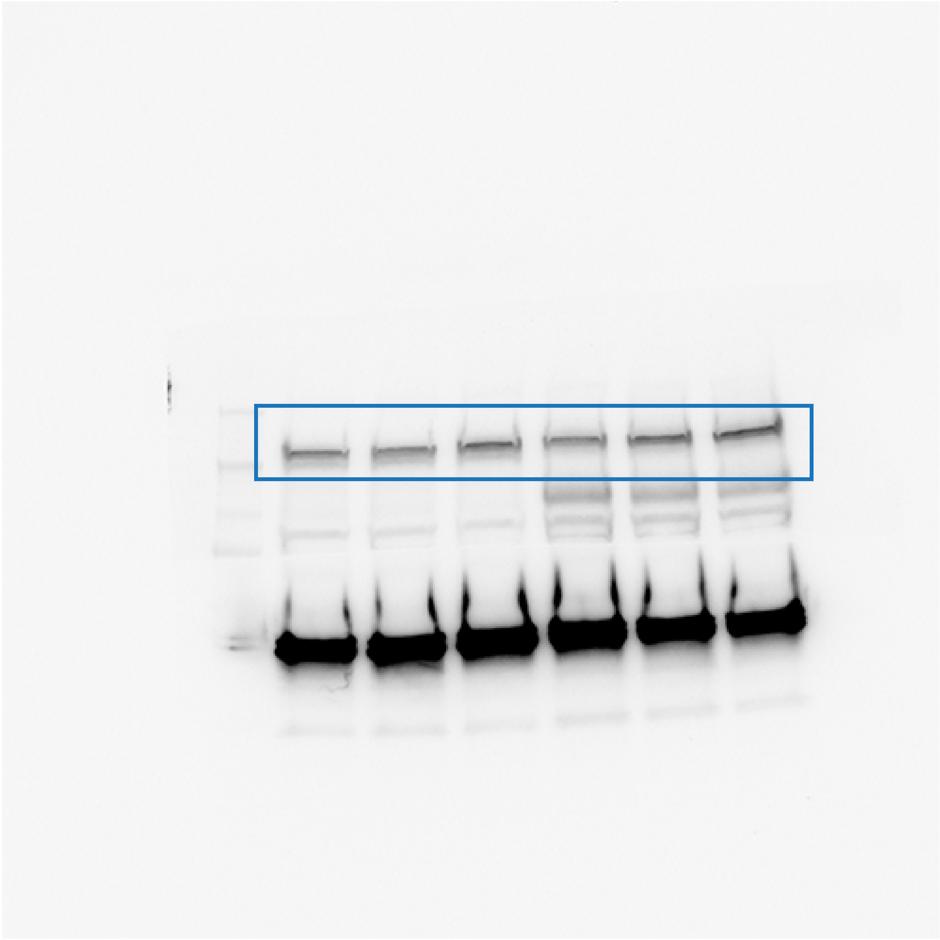

ERK

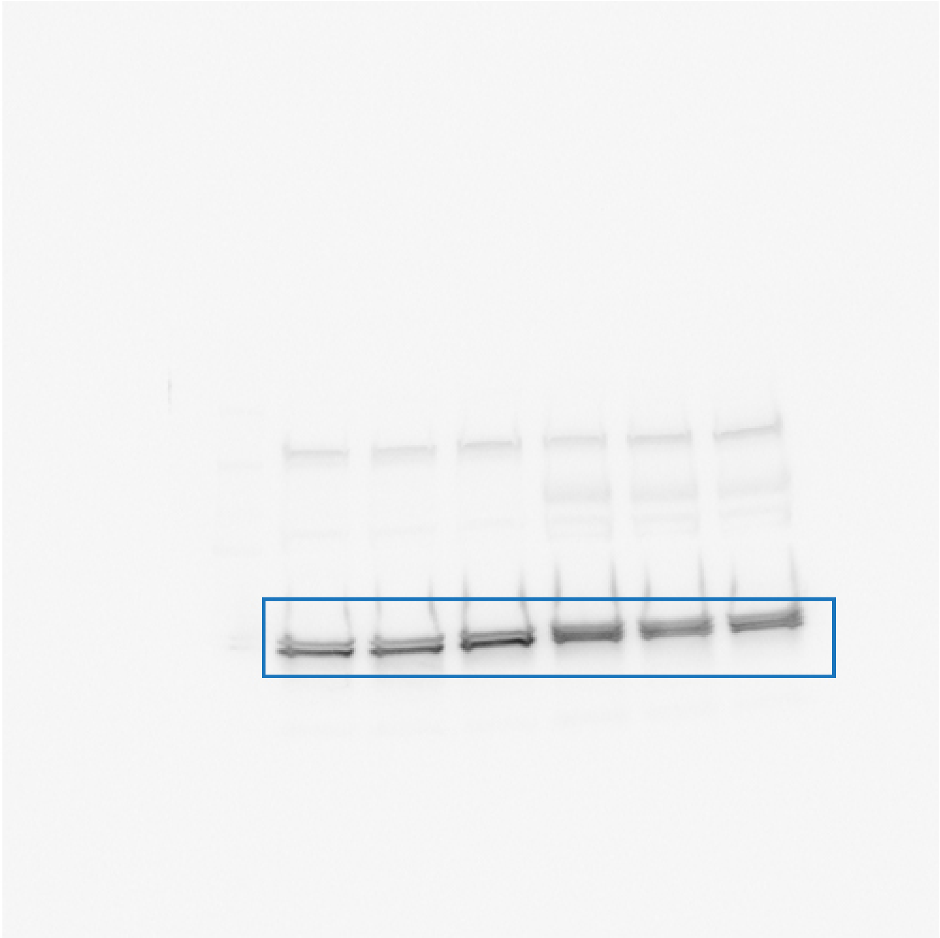

Supplementary Figure 4: Uncropped and unprocessed Western blots for Figure 1.

Uncropped Western blots shown for representative Figures 1a, 1c and 1e. The blue boxes denote regions of interest shown in the representative figures. **Figure 1a:** Blots were first probed for pERK and then probed for ERK and, subsequently, α-Tubulin loading control on the same blot. ERK and α-tubulin are shown on the same blot given their similar size, with α-Tubulin bands shown above ERK bands. **Figure 1c:** Blots were first probed for pEGFR and pERK and then respectively probed for EGFR and ERK. α-Tubulin loading control was probed last.

Figure 2

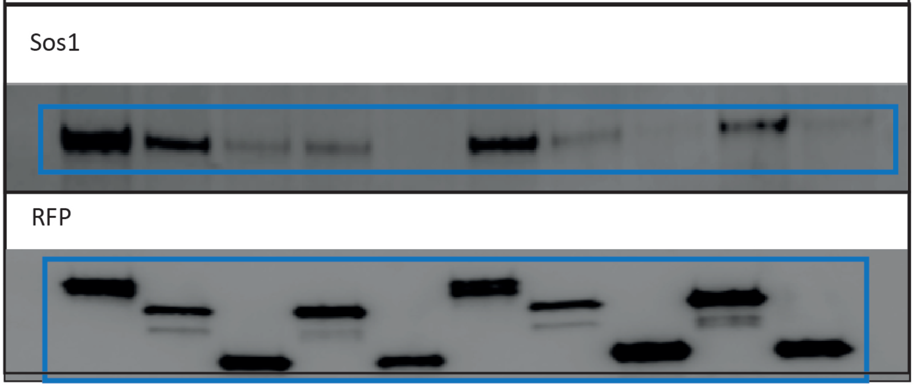

Figure 3a

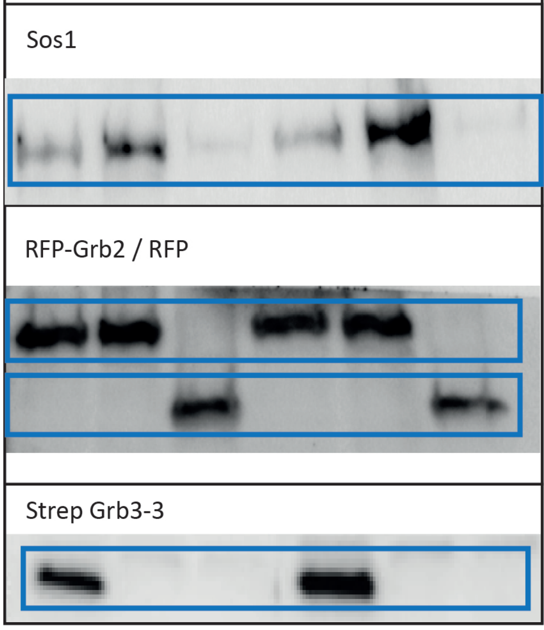

Figure 3b

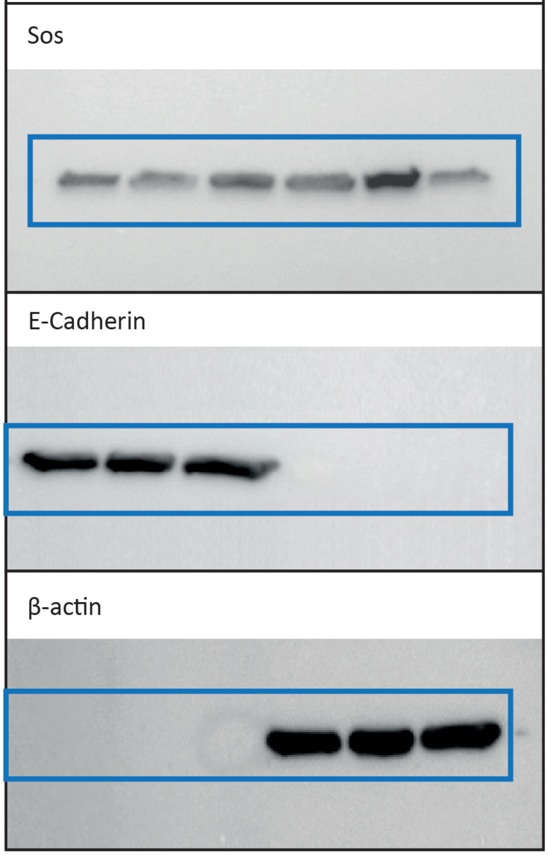

Extended Data Figure 1

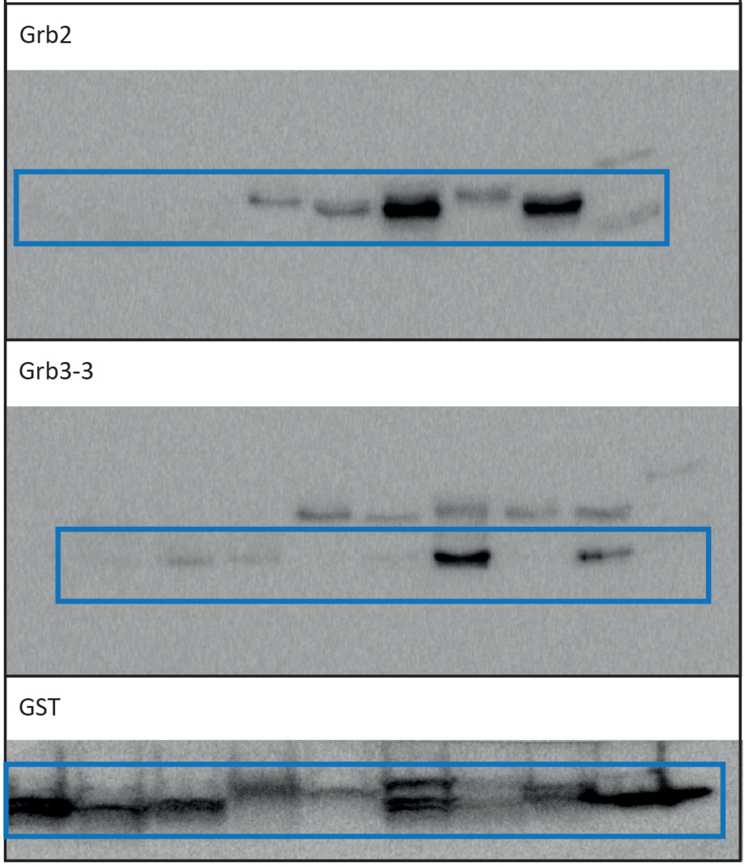

Figure 4

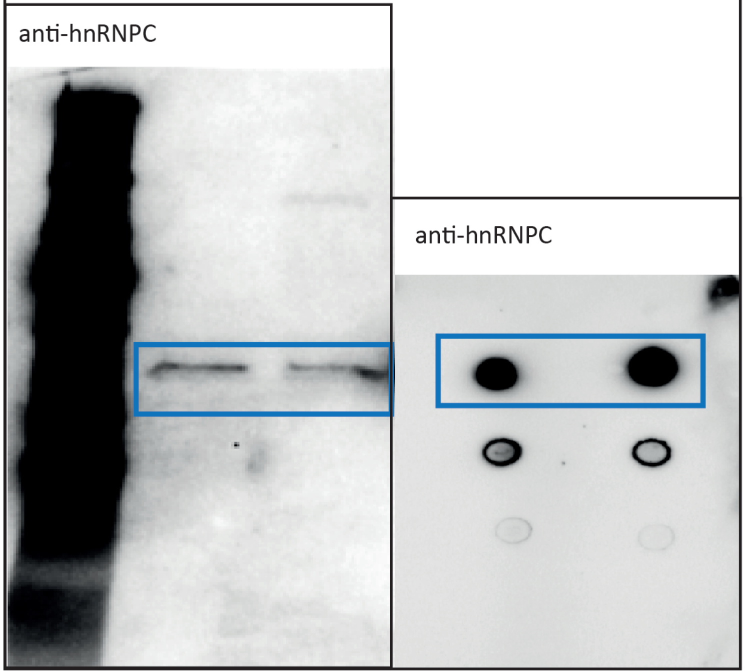

Supplementary Figure 5: Uncropped and unprocessed Western blots for Figures 2, 3 and 4, as well as Supplementary Figure 1

Uncropped Western blots shown for representative **Figures 2, 3, 4** and **Supplementary Figure 1**. Blue boxes denote regions of interest shown in the representative figure.

**Sos domain**	**Peptide name**	**Sequence**
PH	Sos-PH-1	MICCKSNHGQ**PRLP**GASNAEYRLK
CDC25	Sos-CDC25-2	DHYKKYLAKLRSINP**PCVP**FFGIYLTNILKTE
	Sos-CDC25-3	TDYLFNKSLEIEPRNPKPL**PRFPKK**YSYPLKSPGVRPSNPRPGTMRHPTPLQQEP
	Sos-CDC25-4	ESETESTASA**PNSPRTPLTPPP**ASGASSTTDVCSVFDSDHSSPFHSSNDTVFIQVTLPHGPRSASVSSISL
Proline-rich C-terminus tail	Sos-CTail-5	TKGTDEVPV**PPPVPPRRRP**ESAPAESSPSKIM
	Sos-CTail-6	SKHLDS**PPAIPPRQP**TSKAYSPRYSISDRTSISDPPESPPLLPPREPVRTPDVFSSSP
	Sos-CTail-7	LHLQPPPLGKKSDHGNAFF**PNSPSPFTPPPPQTP**SPHGTRRHLPSPPLTQEVDLHSI
	Sos-CTail-8	EVDLHSIAG**PPVPPR**QSTSQHI**PKLPPK**TYKREHTHPSMH

Supplementary Table 1: Amino acid sequences of Sos proline-rich peptides by the domain from which they are isolated. Proline-rich motifs are highlighted in red and by bold, underling type face. NB. Peptide Sos-CTail-8 contains two PXXP motifs. However, the proximity of these means that they are unlikely to sustain two individual SH3 domain binding sites.

Splicing factor	Recognised sequence	Sequence position
ELAVL1	UUUUU	5-9 or 6-10
HNRNP C	UUUUU(U)<br>UUUUUG	5- 9 or 10<br>6-10
KHDRBS1	UUUUUU<br>(CA)AAAU	5-10<br>13-18 or 15-18
HNRNP G	AAAAU<br>AUCCCC	14-18<br>17-22
KHDRBS3	AAAU	15-18 or 39-42
HNRNP E2	UCCCCA	18-23
SRSF9	AGGCA	30-34
SRSF2	AGAAG	34-38
SRSF10	(G)AAGAA	35-40 or 36-40
Tra2alpha	AAGAA	36-40
HNRNP A1	CUUAG	44-48
ELAVL2	CUUUC	70-74
TIA-1	CUUUC	70-74
MBNL1	AUGCUU<br>GCGCUC	41-46<br>93-98
HNRNP F	UGGGG<br>GGGGC<br>UGGGG<br>GGGGA	64-68<br>65-69<br>100-104<br>101-105
HNRNP H1, H2, H3	AAGAA<br>UGGGG<br>GGGGC<br>AGAGA<br>UGGGG<br>GGGGA	36-40<br>64-68<br>65-69<br>82-86<br>100-104<br>101-105
HNRNP I	CUUUCUU<br>CUCUCU<br>UCUCU	70-76<br>113-118<br>114-118
SRSF5	ACAGC	52-56

Supplementary Table 2: Splicing factors predicted via *in silico* analysis to bind to sequences located within *GRB2* exon 4 using SpliceAid2. Around half of the identified proteins are constituents of the hnRNP family. SR proteins are also strongly represented.

In-silico prediction		Experimental validation (number of peptides)		
Splicing factor	RNA oligonucleotide containing binding site	Oligo 1 (1-50)	Oligo 2 (53-103)	Oligo 3 (104-123)
ELAVL1	Oligo1	11	5	3
HNRNP C	Oligo 1	10	0	1
KHDRBS1	Oligo 1	2	0	1
HNRNP G	Oligo 1	8	0	7
KHDRBS3	Oligo 1	0	0	0
HNRNP E2	Oligo 1	9	12	4
SRSF9	Oligo1	0	4	12
SRSF2	Oligo1	4	4	4
SRSF10	Oligo1	0	5	0
Tra2alpha	Oligo1	0	3	3
HNRNP A1	Oligo1	24	19	32
ELAVL2	Oligo2	0	0	0
TIA-1	Oligo2	13	2	3
MBNL1	Oligo1& 2	0	0	0
HNRNP F	Oligo2	6	0	4
HNRNPH1	Oligo1& 2	18	1	16
HNRNPH2	Oligo1& 2	11	1	8
HNRNPH3	Oligo1& 2	4	0	2
HNRNP I	Oligo 2 & 3	29	11	7
SRSF5	Oligo 2	2	0	2

Supplementary Table 3: Experimental validation of proteins binding to *GRB2* exon 4. Splicing factors predicted to bind to exon 4 of *GRB2* by SpliceAid2 are shown, as are the RNA oligonucleotide that contain the consensus binding site for each predicted splicing factor. The number of peptides corresponding to each protein that

were identified by mass spectrometry analysis of each RNA oligonucleotide is shown. Only proteins with two or more matching peptides were considered to have bound.

Oligo/SpliceAid	hnRNP/SR proteins	
	Number	Names
Oligo1, Oligo2, Oligo3, SpliceAid2	2	SRSF2, hnRNPA1
Oligo1, Oligo2, Oligo3	10	hnRNPL, hnRNPK, hnRNPQ, hnRNPUL1, hnRNPA2B1, hnRNPA3, hnRNPR, hnRNPA0, hnRNPAB, hnRNPDL
Oligo1, Oligo3, SpliceAid2	3	hnRNPH1, hnRNPF, hnRNPH2
Oligo2, Oligo3, SpliceAid2	1	SRSF9
Oligo1, Oligo3	8	hnRNPM, SRSF3, hnRNPD, hnRNPU, SRSF4, SRSF7, SRSF1, SRSF6
Oligo1, SpliceAid2	3	hnRNPC, SRSF5, hnRNPH3
Oligo2, SpliceAid2	1	SRSF10
Oligo1	2	hnRNPUL2, hnRNPLL
Oligo2	3	hnRNP H, hnRNPD0, SRSG7
SpliceAid2	3	hnRNPE2, hnRNPI, hnRNPG

Supplementary Table 4: A list of hnRNP/SR proteins identified by mass spectrometry (Oligo1, Oligo2, Oligo3) and predicted to bind by SpliceAid2.

Output type	Output
rMATS ID	27123
Gene ID	ENSG00000177885**.9**
Gene Name	GRB2
Chromosome	17
Gene Strand	-
Exon Start	73321978
Exon End	73322101
Upstream Exon Start	73317875
Upstream Exon End	73317908
Downstream Exon Start	73328780
Upstream Exon End	73328878
Inclusion Counts hnRNPC shRNA	198, 190
Skipping Counts hnRNPC shRNA	1, 5
Inclusion Counts Control	291, 364
Skipping Counts Control	0, 0
Length of Inclusion Form (used for normalisation)	197
Length of Skipping Form (used for normalisation)	99
P Value	0.008984227
False Discovery Rate (calculated from P value)	0.108942659
Inclusion Level for Sample 1	0.99, 0.95
Inclusion Level for Sample 2	1.0, 1.0
Inclusion Level Difference	-0.03

Supplementary Table 5: rMATs analysis of significantly different splicing events for hnRNPC knockdown and control experiments.
