# Supplementary material for: The Grb2 splice variant, Grb3-3, is a negative regulator of RAS activation
Source: Commun Biol. 2022 Sep 28;5:1029. doi: 10.1038/s42003-022-03985-7 (PMC9519967; doi:10.1038/s42003-022-03985-7)
Supplement: Supplementary file 3 — Description of Additional Supplementary Files [file 42003_2022_3985_MOESM3_ESM.pdf]

Description of Additional Supplementary Files

File name: Supplementary Data 1
Description: Mass spectral raw data.

File name: Supplementary Data 2
Description: Identification Numbers of Newly Generated Plasmids – Deposited in Addgene Database.

File name: Supplementary Data 3
Description: All raw data used to generate graphs for Fig. 1b, Fig. 1d.i, 1d.ii, Fig. 1e, Fig. 1f, Fig. 3e, Fig. 3g, Fig. 4d, Fig. 4e.
