# Supplementary material for: The Grb2 splice variant, Grb3-3, is a negative regulator of RAS activation
Source: Commun Biol. 2022 Sep 28;5:1029. doi: 10.1038/s42003-022-03985-7 (PMC9519967; doi:10.1038/s42003-022-03985-7)
Supplement: Supplementary file 5 — Supplementary Data 2 [file 42003_2022_3985_MOESM5_ESM.docx]

Identification Numbers of Newly Generated Plasmids – Deposited in Addgene Database.

| **Plasmid** | **Description/Experimental Purpose** | **ID** |
| --- | --- | --- |
| pET28a-SH2 [Edit] | Express His-tagged Grb3-3 SH2 domain [Edit] | 190908 |
| pET28a-SH2Δ40CSH3 [Edit] | Express His-tagged Grb3-3 with truncated N-terminal SH3 domain [Edit] | 190907 |
| pET28a-NSH3SH2Δ40 [Edit] | Express His-tagged Grb3-3 with truncated C-terminal SH3 domain [Edit] | 190906 |
| pET28a-Grb3-3 [Edit] | Express His-tagged Grb3-3 in bacterial cells [Edit] | 190905 |
| pET28a-Grb2 [Edit] | Express His-tagged Grb2 in bacterial cells [Edit] | 190904 |
