# Supplementary material for: The Grb2 splice variant, Grb3-3, is a negative regulator of RAS activation
Source: Commun Biol. 2022 Sep 28;5:1029. doi: 10.1038/s42003-022-03985-7 (PMC9519967; doi:10.1038/s42003-022-03985-7)
Supplement: Supplementary file 7 — Reporting Summary [file 42003_2022_3985_MOESM7_ESM.pdf]

nature research

Corresponding author(s): John E. Ladbury

Last updated by author(s): July 18, 2022

Reporting Summary

Nature Research wishes to improve the reproducibility of the work that we publish. This form provides structure for consistency and transparency in reporting. For further information on Nature Research policies, see our Editorial Policies and the Editorial Policy Checklist.

Please do not complete any field with "not applicable" or n/a. Refer to the help text for what text to use if an item is not relevant to your study.
For final submission: please carefully check your responses for accuracy; you will not be able to make changes later.

Statistics

For all statistical analyses, confirm that the following items are present in the figure legend, table legend, main text, or Methods section.

n/a	Confirmed	
☐	☒	The exact sample size (n) for each experimental group/condition, given as a discrete number and unit of measurement
☒	☐	A statement on whether measurements were taken from distinct samples or whether the same sample was measured repeatedly
☒	☐	The statistical test(s) used AND whether they are one- or two-sided<br>*Only common tests should be described solely by name; describe more complex techniques in the Methods section.*
☒	☐	A description of all covariates tested
☒	☐	A description of any assumptions or corrections, such as tests of normality and adjustment for multiple comparisons
☒	☐	A full description of the statistical parameters including central tendency (e.g. means) or other basic estimates (e.g. regression coefficient) AND variation (e.g. standard deviation) or associated estimates of uncertainty (e.g. confidence intervals)
☒	☐	For null hypothesis testing, the test statistic (e.g. F, t, r) with confidence intervals, effect sizes, degrees of freedom and P value noted<br>*Give P values as exact values whenever suitable.*
☒	☐	For Bayesian analysis, information on the choice of priors and Markov chain Monte Carlo settings
☒	☐	For hierarchical and complex designs, identification of the appropriate level for tests and full reporting of outcomes
☒	☐	Estimates of effect sizes (e.g. Cohen's d, Pearson's r), indicating how they were calculated

Our web collection on statistics for biologists contains articles on many of the points above.

Software and code

Policy information about availability of computer code

Data collection	N/A
Data analysis	N/A

For manuscripts utilizing custom algorithms or software that are central to the research but not yet described in published literature, software must be made available to editors and reviewers. We strongly encourage code deposition in a community repository (e.g. GitHub). See the Nature Research guidelines for submitting code & software for further information.

Data

Policy information about availability of data

All manuscripts must include a data availability statement. This statement should provide the following information, where applicable:

- Accession codes, unique identifiers, or web links for publicly available datasets
- A list of figures that have associated raw data
- A description of any restrictions on data availability

For all experiments, data were analysed using ANOVA (GraphPad, Prism 9). P-values ≤0.05 were considered statistically significant.

Field-specific reporting

Please select the one below that is the best fit for your research. If you are not sure, read the appropriate sections before making your selection.

☒ Life sciences ☐ Behavioural & social sciences ☐ Ecological, evolutionary & environmental sciences

For a reference copy of the document with all sections, see nature.com/documents/nr-reporting-summary-flat.pdf

Life sciences study design

All studies must disclose on these points even when the disclosure is negative.

Sample size	For all experiments, data were analysed using ANOVA (GraphPad, Prism 9). P-values ≤0.05 were considered statistically significant.
Data exclusions	N/A
Replication	Experiments were repeated as described in Figure Legends using n value
Randomization	N/A
Blinding	N/A

Reporting for specific materials, systems and methods

We require information from authors about some types of materials, experimental systems and methods used in many studies. Here, indicate whether each material, system or method listed is relevant to your study. If you are not sure if a list item applies to your research, read the appropriate section before selecting a response.

Materials & experimental systems

n/a	Involved in the study	
☐	☒	Antibodies
☐	☒	Eukaryotic cell lines
☒	☐	Palaeontology and archaeology
☒	☐	Animals and other organisms
☒	☐	Human research participants
☐	☒	Clinical data
☒	☐	Dual use research of concern

Methods

n/a	Involved in the study	
☒	☐	ChIP-seq
☒	☐	Flow cytometry
☒	☐	MRI-based neuroimaging

Antibodies

Antibodies used	Antibodies raised against phospho-p44/42 MAPK (ERK1/2) (Thr202/Tyr204) (#9101), p44/42 MAPK (ERK1/2) (#9102), α-Tubulin (11H10) (#2125), phospho-EGF receptor (Tyr1068) (D7A5) (#3777), EGF receptor (D38B1) XP® rabbit mAb 1:1000 (#4267), GRB2 rabbit antibody (#3972), E-Cadherin (24E10) (#3195), β-Actin (13E5) (#4970), hnRNP A0 (#4046), RAS (27H5) (#3339), and GST (26H1) (#2624), (#7076) were purchased from Cell Signalling, as was an anti-rabbit IgG HRP-linked antibody (#7074). An anti-RFP rabbit antibody (ab62341) was purchased from Abcam and an anti-SOS1 mouse antibody (WH0006654M1) purchased from Sigma-Aldrich. DH5α
Validation	Antibodies validated as per manufacturers statement(s)

Eukaryotic cell lines

Policy information about cell lines

Cell line source(s)	Mammalian cells were maintained as sub-confluent cultures at 37°C with 5% carbon dioxide (CO2). When not passaged, 50% media changes were undertaken at two-three day intervals. Human colorectal adenocarcinoma Caco-2 (HTB-37™) cells, the highly-transfectable human embryonic kidney (HEK293T; CRL-3216™) cell line and the human cervix adenocarcinoma HeLa (CCL-2™) cell line were purchased from American Type Culture Collection (ATCC; Virginia, USA).
Authentication	Cells were not authenticated expressly for this work. However, we periodically authenticate all lab cell lines.
Mycoplasma contamination	All cells were tested for, and found to be free of, mycoplasma contamination using the LookOut Mycoplasma Detection kit (Thermo Fisher Scientific) applied at monthly intervals.

Commonly misidentified lines (See ICLAC register)	N/A

Clinical data

Policy information about clinical studies

All manuscripts should comply with the ICMJE guidelines for publication of clinical research and a completed CONSORT checklist must be included with all submissions.

Clinical trial registration	N/A
Study protocol	The use of patient samples was approved by the National Research Ethics Service (London – Bloomsbury Research Ethics Committee; 12/LO/1217).
Data collection	N/A
Outcomes	N/A

This checklist template is licensed under a Creative Commons Attribution 4.0 International License, which permits use, sharing, adaptation, distribution and reproduction in any medium or format, as long as you give appropriate credit to the original author(s) and the source, provide a link to the Creative Commons license, and indicate if changes were made. The images or other third party material in this article are included in the article's Creative Commons license, unless indicated otherwise in a credit line to the material. If material is not included in the article's Creative Commons license and your intended use is not permitted by statutory regulation or exceeds the permitted use, you will need to obtain permission directly from the copyright holder. To view a copy of this license, visit http://creativecommons.org/licenses/by/4.0/
